# The study of pubertal stage and age of menarche in girls in Isfahan province, Iran

**DOI:** 10.1186/s12887-024-05212-0

**Published:** 2025-01-31

**Authors:** Nafiseh Mozafarian, Mahin Hashemipour, Mohammad Reza Maracy, Masoumeh Pourrajab, Razieh Omidi, Roya Kelishadi

**Affiliations:** 1 Child Growth and Development Research Center, Research Institute for Primordial Prevention of Noncommunicable Disease, Isfahan University of Medical Sciences, Isfahan, Iran; 2 Metabolic Liver Disease Research Center, Isfahan University of Medical Sciences, Isfahan, Iran; 3 Isfahan Endocrine and Metabolism Research Center, Isfahan University of Medical Sciences, Isfahan, Iran; 4 Department of Biostatistics and Epidemiology, School of Public Health, Isfahan University of Medical Sciences, Isfahan, Iran; 5 Environment Research Center, Research Institute for Primordial Prevention of Non- communicable Disease, Isfahan University of Medical Sciences, Isfahan, Iran; 6 Isfahan Provincial Education and Training Organization, Isfahan, Iran; 7 Health Center of Isfahan Province, Isfahan University of Medical Sciences, Isfahan, Iran

**Keywords:** Puberty, Menarche, Thelarche, Pubarche, Children and adolescent

## Abstract

**Background:**

The onset age of pubertal changes varies widely among girls and has undergone changes over time around the world. This study aimed to determine the age of onset of puberty in healthy Iranian girls living in Isfahan province.

**Methods:**

This cross- sectional study was conducted among 13,886 students aged 6–18 years. They were selected by multistage random cluster sampling from urban and rural areas of Isfahan province of Iran. Pubertal stage was determined according the 5-level Tanner stages proposed by Marshall and Tanner. Trained physicians determined the breast development through both visual inspection and palpation, in addition, mothers assessed their daughter’s pubic hair growth using Tanner’s Sexual Maturation Scale (SMS) by comparison to standard pictures. The data were analyzed by the status quo method. Probit regression analysis was used to evaluate the median age of onset for each characteristic including menarche, breast and pubic hair.

**Results:**

A total of 13,886 healthy girls with mean age (SD) of 11.97 (2.9) were studied. Overall 6968 (50.2%) girls experienced menstruation with a median (95%CI) age of 12.05(11.88–12.21) years at its onset. The median age for onset of breast development was 9.89 (95% CI: 9.77 − 10.02) years and for onset of pubic hair development was 10.14 (95% CI: 9.88– 10.39) years. The 3rd percentile for breast stage 2 (B2) and pubic hair stage 2 (PH2) was 6.85 and 6.75 years, respectively, and the 97th percentile for B2 and PH2 was 12.94and 13.54 years, respectively.

**Conclusion:**

According to our findings, the onset of puberty before 6.85 years should be considered as precocious puberty for Iranian girls. Our study indicated the secular trend toward earlier menarche in girls. Nationally representative surveys and longitudinal studies are necessary to determine the exact age of puberty for future international comparisons.

## Introduction

Puberty is a critical stage of development during which secondary sexual characteristics are developed and the capable of reproduction is created in each individual [1].The physical changes usually began from 8 to 13 years in girls [2]. Puberty milestones in girls include thelarche (onset of breast development), pubarche (onset of pubic hair development) and menarche (first menstruation). Generally, the first and last signs of puberty are thelarche and menarche, respectively.

The age of onset of pubertal changes varies widely among girls around the world and has undergone changes over time. In addition to genetic or ethnic factors, environmental factors such as geographical location, nutrition and socioeconomic conditions can also affect the age of puberty [3–5].

Decreasing trend in timing of puberty is observed in many countries in recent decades, particularly in girls [6–10].The mean age at menarche(AAM) has decreased from 15 to 16 to 12–13 during the last two centuries in Europe [5, 11].Although genetic factors remain relatively constant, the trend may be related to improved health and nutritional status over time [10, 12–15].

Early puberty timing has several adverse health effects including an increased future risk of depression, anxiety, diabetes, heart diseases, cardiovascular disease and breast cancer in adult life [16–18]. Furthermore, adolescent girls with earlier pubertal onset may experience psychosocial difficulties such as more risk-taking behaviors, earlier sexual activity, substance use problems, low academic achievement [19, 20].

Several studies have been conducted to evaluate age at menarche in our country. In 2014, a meta-analysis of 44 studies reported that the mean (95% CI) age at menarche among Iranian girls was 12.81 (95% CI: 12.56–13.06) years [21]. To our knowledge, a few puberty studies have been done to assess the age of different stages of puberty in our country [22–26]. A study conducted among 3192 girls during 2005–2006 in Isfahan, Iran. The median AAM, B2 and PH2 was 12.65, 10.14 and 10.78 years, respectively [23]. In 2006, a national study conducted in 20 provinces in Iran by Motlagh et al., result showed the difference in the age of puberty among girls living in different provinces of our country [24].

In order to make clinical judgments and prevent over-treatment on patients, provide appropriate patient education and also assessment of secular trend, it is necessary to periodically provide the normal limits for stages of puberty in each population. In this survey, we aimed to determine the timing of puberty in a group of Iranian girls living in Isfahan province.

## Methods

### Study population

This cross-sectional study was conducted among 13,886 students aged 6–18 years. They were selected by multistage random cluster sampling from urban and rural areas of Isfahan province of Iran from 2021 to 2023. The first, stratification was done in the province base on the geographic regions (north, south, east, west and central). Then, 12 cities including Najafabad, Ardestan, Natanz, Mobarakeh, Golpayegan, Falavarjan, Shahreza, Fereydunshahr, Shahinshahr, Jarqavieh Sofla, Kuhpayeh and Harand city were selected randomly from the different areas of Isfahan province. In addition, the city of Isfahan was selected as the capital of Isfahan province.

In selected cities, private and public schools were also selected randomly. In selected schools, all the students were invited to participate in the study. Participation in the study was voluntary.

The number of students in each city was determined according to the student’s place of residence and level of education (elementary, intermediate and high schools) by the proportionate sampling.

We enrolled healthy girls with Iranian nationality, residence in Isfahan province. If they had a history of chronic diseases, a genetic syndrome and use of medications that affects pubertal development (e.g., GnRH agonist), refusal to participate in this study and with missing information on puberty timing were excluded from the study.

The study protocol was approved by the ethics committee of Isfahan University of Medical Sciences. (Approval code: IR.MUI.MED.REC.1399.176 research project number 398986). The purposes of the study were explained to students and their parents, and they were assured that their information would be kept confidential. Oral and written informed consent was obtained from all eligible students and one of their parents, respectively.

The survey was performed with the cooperation and coordination of Metabolic Liver Diseases Research Center and Child Development Research Center (CDRC) at the Isfahan University of Medical Sciences and department of education in Isfahan province and the Health Center of Isfahan province.

Information was collected by a validated questionnaire, clinical examinations and anthropometric measurements.

### Anthropometric measurements

A trained team of health care measured the weight and height of students using calibrated instruments according to the standard protocols. Students’ weights were measured with minimum clothing and without shoes to the nearest 0.1 kg by an electronic scale and height were measured in barefoot to the nearest 0.1 cm by a non-elastic tape meter. BMI was calculated as weight (kg) divided by height (m^2^). We used the World Health Organization’s specific BMI curves based on height and age to categorize participants’ BMI. BMI less than 5th percentile as underweight; BMI between 5th and 85th percentiles as normal weight; BMI between 85th and 95th percentiles as overweight and BMI greater than the 95th percentile as obese [27].

### Tanner staging

Pubertal development evaluated by a physician or maternal report using Tanner staging. The breast development was only assessed according to the criteria of Marshall and Tanner by trained pediatricians in this study. However, we were not able to clinically evaluate the tanner stage of pubic hair, so mothers assessed their daughter’s pubic hair development using Tanner’s Sexual Maturation Scale. The pubertal stages of breast and pubic hair development are categorized in 5 stages (B1 to B5, PH1 to PH5, respectively). The onset of breast development before age 8 years is commonly considered as precocious puberty globally.

### Clinical tanner stage

A pediatric endocrinologist (MH) trained a number of pediatricians to assess secondary sexual characteristics. The physicians and the pediatric endocrinologist (MH) performed clinical breast tanner staging. The breast development through both visual inspection and palpation was assessed according to the criteria of Marshall and Tanner [28]. When the breast differed in size between the two sides, the highest Tanner stage was used.

### Self-assessment of tanner stages

Tanner’s Sexual Maturation Scale (SMS) is a self-report instrument of puberty [28]. This questionnaire includes a series of photographs or drawings with explanatory text of five stages of pubic hair development (Tanner PH1-5). We previously performed the validity and reliability of the questionnaire against clinical examination. The Cronbach’s alpha of the questionnaires was 0.83. The ICC of the questionnaires was 0.95 (0.92–0.98) [29].

Using the SMS, mothers assessed their daughter’s pubic hair development by selecting one of five photographs showing PH1–PH5. The mothers were asked to complete the questionnaire by themselves or with their daughter.

### Evaluation of menarchal age

Menarche status was assessed during the clinical examination by two questions:1. Have you started having periods? (yes, no) 2. How old were you when you had your first period? The participants were asked to report the year and month of their first period.

### Statistical analysis

The data were analyzed by the status quo method and probit regression. The girls were grouped on 6-month age intervals. For example, the 6.25-year-old girls included girls aged 6 to 6.5 years.

The proportion of girls within each age group who had reached a certain event (menarche, B2-B5 and PH2-PH5) were presented. The median age and percentiles of the onset for each characteristic were calculated using probit modelling. Then, we fitted cumulative frequency curves for each Tanner stage of breast and pubic hair and menarche with the use of probit analysis. The data were analyzed by the SPSS software version 18.0 (SPSS Inc., Chicago, IL, USA).

## Results

A total of 13,886 healthy girls aged 6–18 years with mean age (SD) of 11.97 (2.9) were studied. The prevalence of underweight, overweight and obesity was 9.5%, 13.1% and 15.5% respectively. The number and percentage of girls according age and their distribution in each of the breast and pubic hair stages as well as menarche status are shown in Table 1. About 50.2% of the participants had experienced their first menstruation.

**Table 1:** Distribution of schoolgirls in each age group (year) per pubertal staging and menarche status

Age (y)	Breast stage(*N* = 6560)					Pubic hair stage (*N* = 5193)					Menarche (13886)		
	B1	B2	B3	B4	B5	PH1	PH2	PH3	PH4	PH5	Yes	No	Total
5–6	1(0.03)	0(0.0)	0(0.0)	0(0.0)	0(0.0)	0(0.0)	0(0.0)	0(0.0)	0(0.0)	0(0.0)	0(0.0)	2(0.03)	2
6–7	280(8.2)	11(0.8)	1(0.2)	0(0.0)	0(0.0)	185(7.9)	7(0.7)	0(0.0)	1(0.2)	0(0.0)	0(0.0)	331(4.8)	331
7–8	991(28.9)	51(3.7)	13(2.0)	0(0.0)	1(0.1)	512(21.7)	31(3.3)	1(0.1)	0(0.0)	0(0.0)	0(0.0)	1143(16.5)	1143
8–9	978(28.5)	184(13.2)	26(3.9)	2(0.5)	4(0.6)	573(24.3)	121(12.7)	10(1.4)	3(0.5)	1(0.2)	2(0.03)	1339(19.4)	1341
9–10	633(18.5)	309(22.2)	79(12.0)	29(7.2)	14(2.1)	496(21.1)	189(19.9)	51(7.3)	16(2.6)	3(0.5)	7(0.1)	1238(17.9)	1245
10–11	371(10.8)	387(27.7)	207(31.3)	108(26.7)	79(11.8)	341(14.5)	269(38.3)	166(23.7)	72(11.5)	23(4.1)	120(1.7)	1293(18.7)	1413
11–12	120(3.5)	285(20.4)	170(25.7)	143(35.3)	141(21)	169(7.2)	201(21.1)	201(28.7)	159(25.5)	46(8.2)	472(6.8)	923(13.3)	1395
12–13	37(1.1)	99(7.1)	110(16.6)	83(20.5)	127(18.9)	58(2.5)	76(8.0)	154(22.0)	180(28.8)	115(20.5)	690(13.8)	429(6.2)	1389
13–14	17(0.5)	58(4.2)	40(6.1)	13(3.2)	117(17.4)	14(0.6)	51(5.4)	92(13.1)	108(17.3)	125(22.2)	1418(20.4)	174(2.5)	1592
14–15	0(0.0)	8(0.6)	10(1.5)	7(1.7)	79(11.8)	3(0.1)	2(0.2)	14(2.0)	31(5.0)	90(16)	1407(20.2)	30(0.4)	1437
15–16	0(0.0)	2(0.1)	2(0.3)	12(3.0)	82(12.2)	2(0.1)	4(0.4)	7(1.0)	39(6.3)	103(18.3)	1460(20)	12(0.2)	1472
16–17	0(0.0)	1(0.1)	1(0.2)	7(1.7)	23(3.4)	2(0.1)	0(0.0)	5(0.7)	14(2.2)	49(8.7)	975(14)	5(0.1)	980
17–18	0(0.0)	0(0.0)	1(0.2)	1(0.2)	2(0.3)	0(0.0)	0(0.0)	0(0.0)	1(0.2)	5(0.9)	127(1.8)	0(0.0)	127
18–19	0(0.0)	0(0.0)	1(0.2)	0(0.0)	2(0.3)	0(0.0)	0(0.0)	0(0.0)	0(0.0)	2(0.4)	19(0.3)	0(0.0)	19
Total	3428	1395	661	405	671	2355	951	701	624	562	6967	6919	13,886

Table 1 shows the number and column percent.

**Fig. 1:**
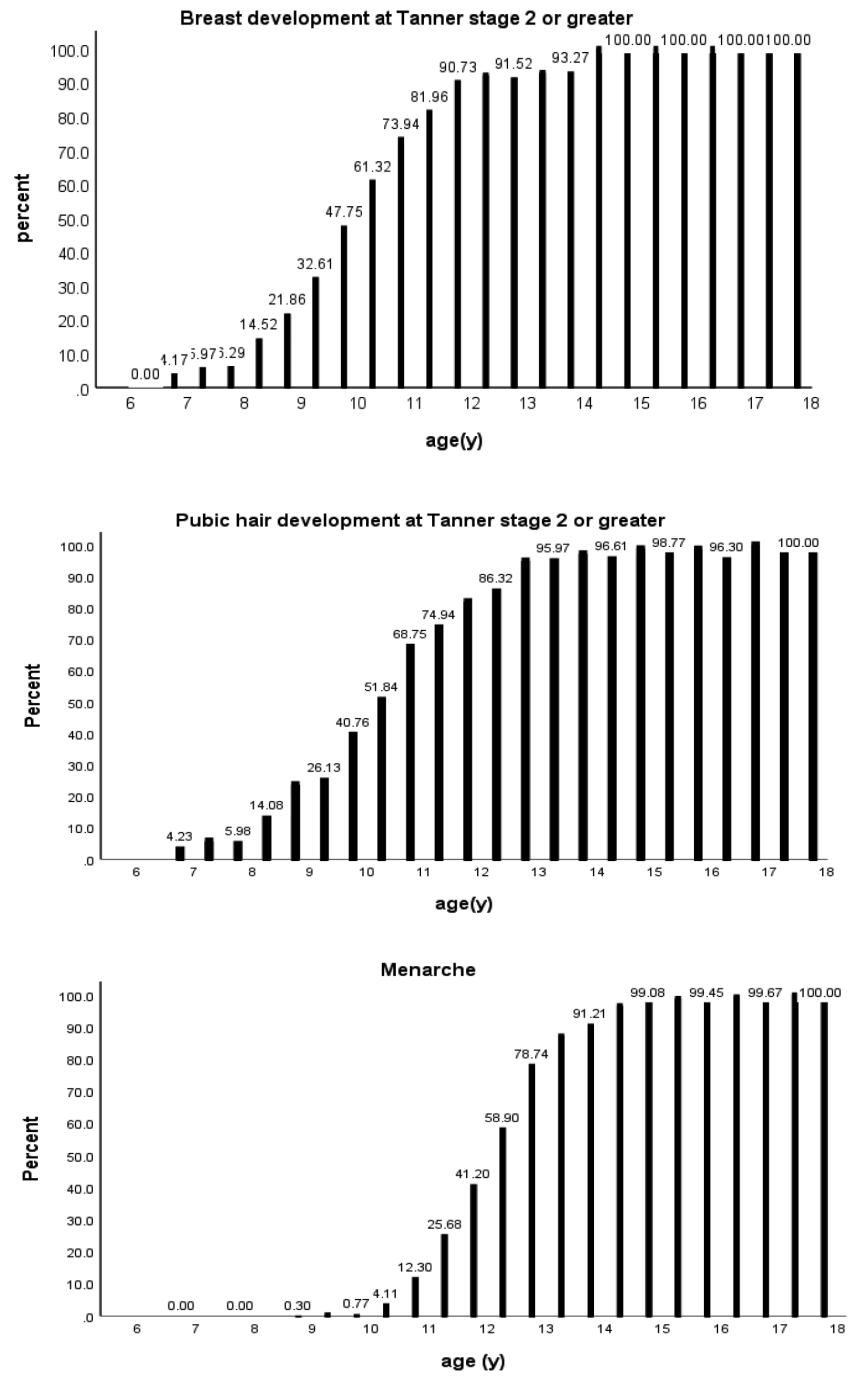
The study of pubertal stage and age of menarche in girls in Isfahan province, Iran

The prevalence of breast and PH development at Tanner stage 2 or greater at each age group are presented in Fig. 1. At age groups 6.75, 7.25 and 7.5 years, 4.17, 5.97 and 6.29% of the girls showed evidence of breast stage 2, respectively. With almost the same prevalence,4.23, 5.76 and 5.98% of the girls had stage 2 pubic hair development at age groups 6.75, 7.25 and 7.5 years, respectively. Only 0.3%(*n* = 2) of girls aged 8.5-9 years had begun menses.

**Fig. 2:**
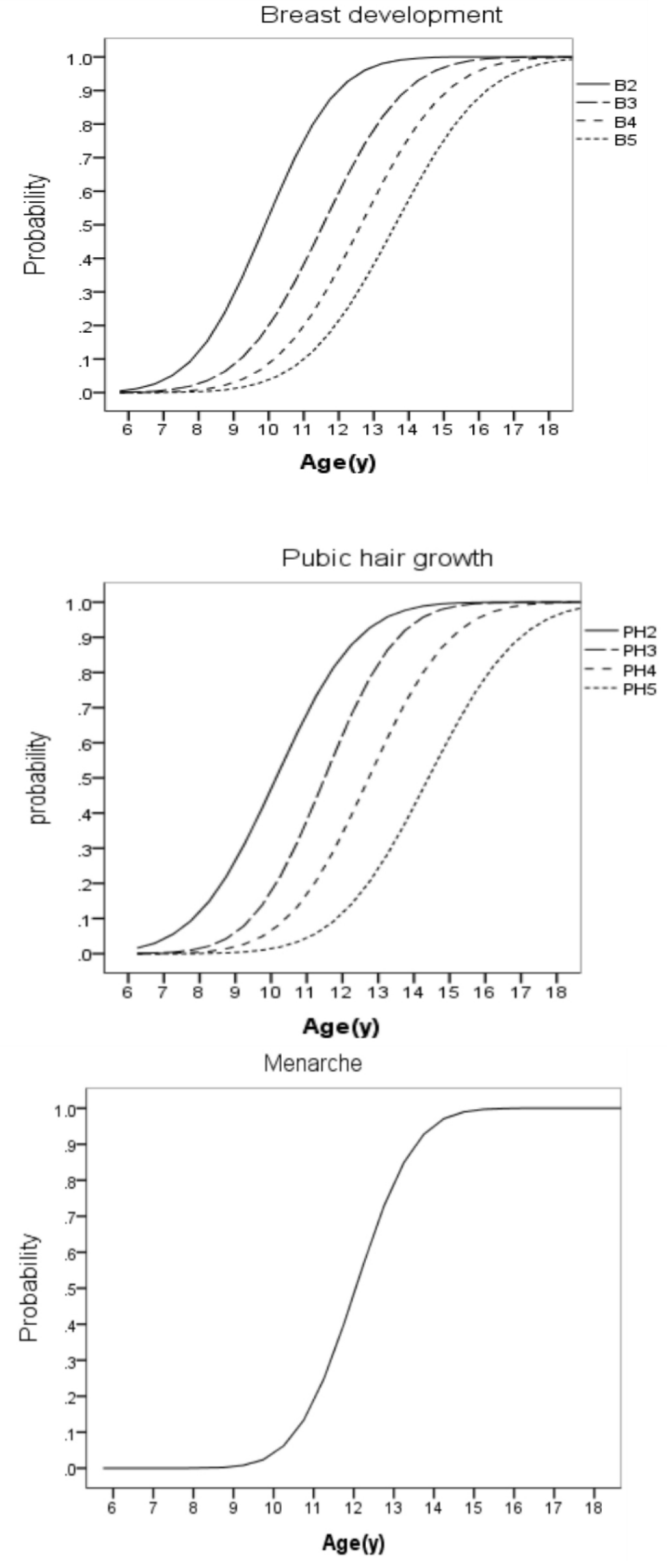
Reference curves for secondary sexual characteristics and menarche in Iranian girls

Results of probit analysis indicated the median ages for attainment of pubertal stages and menarche (Table 2). Overall, 6968 (50.2%) girls experienced menses with a median age(95%CI) of 12.05(11.88–12.21) years at its onset. The median age for onset of breast development was 9.89 (95% CI: 9.77 − 10.02) years. The median age for onset of pubic hair development was 10.14 (95% CI: 9.88–10.39) years. The 3rd percentile for B2 and PH2 was 6.85 and 6.75 years, respectively, and the 97th percentile for B2 and PH2 was 12.94 and 13.54 years, respectively. Figure 2 presents the reference curves for different sexual development.

**Table 2:** Ages of attainment of various pubertal stages and menarche according to probit analysis in the study participants

The pubertal stages and menarche	*N*	Age, y						
		P3	P10	P25	P50(95%CI)	P75	P90	P97
Breast stage 2	3132	6.85	7.82	8.81	9.89(9.77–10.02)	10.99	11.97	12.94
Breast stage 3	1737	8.10	9.20	10.32	11.55(11.31–11.84)	12.79	13.91	15.01
Breast stage 4	1076	9.01	10.16	11.33	12.64(11.86–14.12)	13.94	15.11	16.26
Breast stage 5	671	9.79	11.01	12.24	13.61(13.12–14.29)	14.98	16.21	17.43
Pubic hair stage 2	2838	6.75	7.83	8.92	10.14(9.88–10.39)	11.36	12.45	13.54
Pubic hair stage 3	1887	8.48	9.44	10.42	11.49(11.34–11.67)	12.58	13.55	14.52
Pubic hair stage 4	1186	9.34	10.43	11.52	12.74(12.52–12.99)	13.95	15.05	16.13
Pubic hair stage 5	562	10.64	11.84	13.06	14.41(14.21–14.63)	15.76	16.97	18.17
Menarche	6967	9.86	10.55	11.26	12.05(11.88–12.21)	12.83	13.54	14.24

## Discussion

The current study conducted to provide normative data on the timing of puberty among schoolgirls in Isfahan province. Using probit estimates, our result showed that median age of the onset of B2 and PH2 was 9.89 (95% CI: 9.77 − 10.02) and 10.14 (95% CI: 9.88– 10.39) years, respectively. The median AAM was 12.05 (95% CI: 11.88 − 12.21) years. Our results show that the onset of puberty, especially menstruation, occurs earlier than two decades ago in Isfahan. This suggests a secular trend toward earlier menarche over time.

Median ages at Tanner breast stage 2 have different patterns across time and geographic locations. A recent meta-analysis found that the earliest and latest onset were reported in the United States (8.8–10.3 years) and in Africa (10.1–13.2 years), respectively [30].Only a few studies have been carried out in our country (Table 3). In 2006,the national estimation of the mean age of breast development at stage 2 was 10.10 in Iranian girls [31]. And at provincial level, the earliest and latest onset in 2006 were reported in Ghazvin-Zanjan region (8.97 ± 1.45 years) and in Fars (11.01 ± 1.88 years), respectively [24]. A cross-sectional study during 2005–2006 by Kashani et al. among 3192 girls in Isfahan showed that the median ages of B2 was 10.14 years (8.33–11.95 years) [14]. an earlier age of breast development was observed in our study. The median age at thelarche in the current study was 9.89 (95% CI: 9.77 − 10.02).

**Table 3:** Median age of pubertal development of girls in some studies

Authors	Place/ data collection	Age range	Number	Mean or median(95%CI) Age (year)		
				Age at B2	Age at PH2	Age at menarche
Marshall-Tanner [29]	England/ 1948–69	8–18	192	11.15	11.69	13.47
Aminorroaya [41]	Tehran/1995	7–15	1331	10.6	10.2	12.9
Rabbani [23]	Tehran/2001 to 2004	6–20	4020	10.15	10.48	14.54
Razzaghy-azar [26]	Tehran/2003–2004	6–17	1420	9.74	10.49	12.68
Rabbani [32]	Iran (All provinces)/2006	6–20	7493	10.10	9.83	12.55
Kashani-Hashemipour [24]	Isfahan/2005–2006	6–17	3192	10.14	10.78	12.65
Salak [42]	Isfahan/2006	5.5–17	3204	10.46	11.15	12.56
Saffari [27]	Qazvin /2009–2010	6–16	2240	9.71	9.82	12.52
Current study	Isfahan/2022	6–18	13,886	9.9	10.1	12.05

B: Breast Development; PH: Pubic Hair

In 2006, a national study showed that the mean age at pubic hair stage (P2) was 9.83 among Iranian girls [31]. Pervious cross-sectional studies reported that the mean age at pubic hair stage (PH2) in Tehran(2001–2004) [22], Isfahan (2006) [23] and Qazvin (2010) [26] was 10.48, 10.78 and 9.82 years, respectively. Our result showed that median age of pubarche was 10.14 (95% CI: 9.88– 10.39) years. Pubic hair Tanner stage was not evaluated by the clinical raters in our study because of low acceptability of study participants and their parents. The previous studies used clinical examination so our results may not be directly comparable to them.

The method of self or parent reports is as a technique often used in large-scale epidemiology studies to assess timing of pubertal onset. The methods provide information with less accuracy than clinical assessment and may be prone to misclassification and measurement error. However, we previously found that the maternal assessment using SMS was a valid and reliable tool for assessment of sexual maturity in Iranian adolescents [29].

A national survey among7493 girls aged 10–18 years in 2006 showed that the mean age at onset of menstruation in Iranian girls was 12.55 years [31]. A cross-sectional study during 2001–2004 by Rabbani et al. on 4020 Tehranian girls showed that the mean AAM was at 14.54 years [22]. In 2006,The mean AAM for Isfahani girls was 12.65 years [23].Our data indicated an earliest age of menarche (12.05 years).It declined by 0.6 years during the last two decades in Isfahan. It indicated that age at menarche has trended younger since the last two decades in Isfahan. In our study, a relatively early menarche may be related to the changes in nutritional status and lifestyle and decreases in physical activity during early life. As, children’s nutritional status can affect the timing of puberty [32].

The downward trend of AAM has been observed in many diverse countries. Childhood obesity can be associated with early menarche in girls [33, 34].In this context, several meta-analysis studies have recently suggested that nutritional status during childhood [15] low levels of vitamin D [35]childhood obesity [36, 37], Longer sleep duration [38] and exposure to environmental endocrine disruptors for instance, exposure to di-(2-ethylhexyl)-phthalate (DEHP) and di-n-butyl phthalate (DBP) [39]may be associated with early menarche in girls. Further studies are essential for advancing knowledge about potentially modifiable nongenetic factors and precocious puberty in different racial/ethnic groups.

The age of normal onset of puberty is conventionally defined as the mean age plus or minus two standard deviations of the age of onset of breast stage 2 in any population [40]. Also, the 3rdand 97th percentile age for age at onset puberty can be considered as precocious and delayed puberty, respectively.

The age limits of normal puberty were previously provided by Marshall and Tanner on 192 British girls from the lower socio-economic sector in 1969. As Marshall and Tanner presented, beginning at the earliest age of 8 years is defined as precocious [28]. Today, many clinicians often use the traditional age cut-offs to monitor the onset of puberty in girls. However, in previous studies this cut-off point varied from 6.2 to 7.5 years to define precocious in Iranian girls [22, 23, 25, 26, 31].

A recent meta-analysis of 30 studies showed a downward trend in the age at thelarche by 3 months per decade from 1977 to 2013 [30]. Results from the current study also suggested that *< 10%* of girls experienced onset of puberty before age 8. The 3rd and 10th percentile age for B2 were 6.81and 7.81, respectively. So, the classical definition for pubertal onset may be outdated in many countries and lead to unnecessary test and treatment in healthy girls. Current and relevant data are necessary to define precocious and delayed puberty in any population.

### Strengths and limitations of this study

The strength of our study is that we conducted a large school- based study to evaluate age of puberty of girls using parent- reported information and the clinical examinations by highly trained staff. We also tried to select the participants from different cities of Isfahan province to cover various characteristics of lifestyle, socioeconomic and environments to obtain a large representative sample of Isfahani girls in a school setting. So, generalizability of the study may be increased.

Our study has some limitations. The crosssectional design of the survey is its’ major limitation by which the exact age of the onset of puberty as well as the speed of the consecutive stages of puberty cannot be accurately determined.

On the other hand, interobserver variability should be considered in the measurement of breast development. However, about 70% of the clinical examinations were performed by one senior endocrinologist (MH) in our study. And only about 30% of the examinations were performed by the trained pediatricians.

So, the interobserver variability may be minor. On the other hand, proper training of physicians by the endocrinologist may limit the errors, differences in training, in the physician-assessments. In addition, we were not able to clinically evaluate the tanner stage of pubic hair. Thus, information bias is possible.

## Conclusion

Our data present the norms of the timing of puberty on schoolgirls in Isfahan province. Puberty symptoms, especially menstruation, were obtained at a slightly lower age than earlier studies in our country. Nationally representative surveys and prospective studies to determine the exact age and tempo of puberty are necessary.

## Data Availability

No datasets were generated or analysed during the current study.
